# Ultra-Stable Inorganic Mesoporous Membranes for Water Purification

**DOI:** 10.3390/membranes14020034

**Published:** 2024-01-27

**Authors:** Ralph A. Bauer, Minghui Qiu, Melissa C. Schillo-Armstrong, Matthew T. Snider, Zi Yang, Yi Zhou, Hendrik Verweij

**Affiliations:** 1 Global Research and Development Inc., 539 Industrial Mile Road, Columbus, OH 43228, USA; 2 State Key Laboratory of Materials-Oriented Chemical Engineering, College of Chemical Engineering, Nanjing Tech University, Nanjing 210009, China; qiumh_1201@njtech.edu.cn; 3 Minnesota Mining and Manufacturing Company, 2501 Hudson Road, Maplewood, MN 55144, USA; mschillo@mmm.com; 4 Carbon-Carbon Advanced Technologies, 4704 Eden Road, Arlington, TX 76001, USA; msnider@c-cat.net; 5 Department of Materials Science and Engineering, The Ohio State University, 140 W 19th Ave, Columbus, OH 43210, USA; 6 Quantumscape, 1730 Technology Drive, San Jose, CA 95110, USA

**Keywords:** inorganic membranes, nanofiltration, ultrafiltration, water purification

## Abstract

Thin, supported inorganic mesoporous membranes are used for the removal of salts, small molecules (PFAS, dyes, and polyanions) and particulate species (oil droplets) from aqueous sources with high flux and selectivity. Nanofiltration membranes can reject simple salts with 80–100% selectivity through a space charge mechanism. Rejection by size selectivity can be near 100% since the membranes can have a very narrow size distribution. Mesoporous membranes have received particular interest due to their (potential) stability under operational conditions and during defouling operations. More recently, membranes with extreme stability became interesting with the advent of in situ fouling mitigation by means of ultrasound emitted from within the membrane structure. For this reason, we explored the stability of available and new membranes with accelerated lifetime tests in aqueous solutions at various temperatures and pH values. Of the available ceria, titania, and magnetite membranes, none were actually stable under all test conditions. In earlier work, it was established that mesoporous alumina membranes have very poor stability. A new nanofiltration membrane was made of cubic zirconia membranes that exhibited near-perfect stability. A new ultrafiltration membrane was made of amorphous silica that was fully stable in ultrapure water at 80 °C. This work provides details of membrane synthesis, stability characterization and data and their interpretation.

## 1. Introduction

Thin, supported mesoporous inorganic membranes are used in water purification and as intermediate layers to deposit other membranes. They typically consist of oxides such as Al_2_O_3_, CeO_2_, SiO_2_, TiO_2_ and ZrO_2_. Their porosity is around 35%, their pore size is between 2 and 50 nm and their thickness is between 10 nm and 10 μm [1,2,3,4,5]. The membranes are generally present (supported) on thick, permeable macroporous supports [6]. The ultimate liquid transport performance that can be obtained is as follows:

- *f_ℓ_* = 4.4 × 10^−12^ m and *f*_V_ = 1600 L/(m^2^Bar·h) for a nanofiltration membrane with a porosity of 35%, straight, 2 nm pores and a thickness of 10 nm.
- *f_ℓ_* = 2.2 × 10^−11^ m and *f*_V_ = 7900 L/(m^2^Bar·h) for an ultrafiltration membrane with a porosity of 35%, straight, 10 nm pores and a thickness of 50 nm.

*f_ℓ_* = *j*_V_ × *η_ℓ_*/Δ*p* is the mechanical permeance, *f*_V_ = *j*_V_/Δ*p* is the volumetric permeance, *j*_V_ is the volumetric flux, *η_ℓ_* is the liquid viscosity and Δ*p* is the mechanical pressure difference. For the calculation of *f*_V_, a dynamic viscosity of 10^−3^ Pa·s is assumed. The underlying assumptions in the calculation of *f_ℓ_* and *f*_V_ are an incompressible non-slip (laminar) flow, following the Hagen–Poiseuille equation, an absence of support resistance and a minimum thickness that is 5× the pore diameter. The *f*_V_ values of known polymeric nanofiltration membranes range from 0.3 to 1.6 L/(m^2^Bar·h) [7,8,9]; the *f*_V_ values of known organic ultrafiltration membranes are <500 L/(m^2^Bar·h) [10,11,12,13]. This marked difference is associated with a higher porosity and smaller minimum thickness of the inorganic membrane. However, the ultimate values for supported inorganic membranes have not yet been attained. In a recent study of supported ceria membranes with 3 nm pores, *f*_V_ = 43 L/(m^2^·h·bar) and >80% Na^+^ rejection were obtained for a thickness of 200 nm. Further increases in *f*_V_ would require more permeable support structures and the development of more thin-membrane-deposition processes.

By far, the most well-known supported mesoporous inorganic membrane is γ-alumina, which was developed during the Manhattan project for the enrichment of ^235^U [14]. Work on γ-alumina membranes for other applications started in the 1980s [15,16,17]. T.A. Kuzniatsova et al. presented methods to improve the microstructural homogeneity of γ-alumina membranes [5]. M.C. Schillo et al. reported on γ-alumina membranes by means of rapid thermal processing with a near 100% rejection of Ca^2+^ ions [18]. The use of mesoporous titania and zirconia membranes for large-scale industrial and environmental water purification has been widely published [19,20,21,22]. In addition, the possibility of further improvements by making composite structures was studied. Zhu et al. reported a mullite–carbon nanotube (CNT) composite membrane with an average porosity of 56% and a long-term permeance of up to 38.7 L/(m^2^Bar·h). They concluded that the addition of CNTs resulted in a stable, highly porous network and hence high permeability [23]. CNTs consist of pure carbon and can therefore be considered fully water stable under normal conditions. In [23], no data were reported for the water stability of the mullite phase. More recently, the water purification properties of thin, supported graphene oxide (GO) membranes were studied [24,25]. Similar to the case for CNTs, GO can be considered fully water stable. But, since thin CNT and GO membranes require oxidic supports and/or additives, the question of the water stability of oxide membranes remains.

Mesoporous oxides can consist of a single or multiple phases, be amorphous or crystalline and contain cationic mixtures, such as in (Ti,Zr)O_2_ and (Y,Zr)O_2−*δ*_. They can also contain substantial numbers of protons and hydroxide groups and, for this reason, we prefer to indicate γ-Al_2_O_3_ as γ-alumina. An advantage of mesoporous inorganic membranes over their polymeric counterparts is the stability of the porous structure at high pressures, the absence of swelling in nearly all solutions and, presumably, chemical resistance under harsh conditions [26,27,28]. In addition, mesoporous inorganic membranes can be applied on piezoelectric macroporous supports which are also inorganic and can generate ultrasound via the application of an alternating voltage during operation [29]. The ultrasound then breaks up laminar boundary layers and keeps the surface clean. Non-piezoelectric supports can be brought into ultrasonic resonance by the application of external piezoelectric transducers. In any case, the supports must have a simple (tubular) geometry with dimensional tolerances within microns, which is only possible with inorganic structures. While supported inorganic membranes are and will remain more expensive than polymeric membranes, the advantages mentioned above may justify the higher cost.

An important requirement for the practical application of these membranes is operational stability, which includes resistance to contamination (fouling), aging, reactions with the filtration medium and cleaning operations. While is it known that γ-alumina is not stable in aqueous solutions at any pH and even under ambient conditions, the other oxides are generally assumed to be fully stable in aqueous environments with pH values around 7 [30,31,32]. However, their actual stability over longer times, at elevated temperatures and with more extreme pH values is mostly unknown. We ascribe this to (1) observation times of <500 h in most published works, (2) fouling effects that obscure the effect on the actual membrane structure, (3) poorly defined membrane structures and (4) resource limitations. The substantial cost of inorganic water-filtration membranes dictates that their operational lifetimes must be up to several years. Lack of insight in this matter has, thus far, hindered large-scale introduction, particularly for water filtration, high-temperature membrane reactors and membrane distillation. Hence, we decided to develop a method for the accurate and precise determination of the actual long-term solution stability of mesoporous inorganic membranes, which is not available at present.

**Figure 1:**
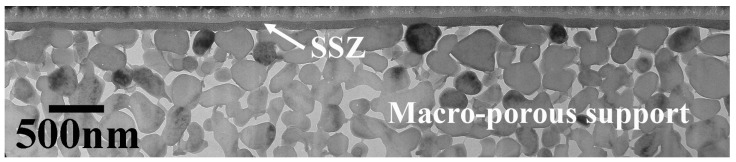
Example of a ~50 nm thick scandium-stabilized zirconia (SSZ) layer on a sintered AKP30 α-Al_2_O_3_ support. This image, which was obtained via transmission electron microscopy, is of a 100 nm thick slab made with focused ion beam milling.

As will be elaborated in the Materials and Methods section, the options for usable stability studies are limited to direct observations of membrane thickness and density using a non-destructive and preferably non-contact method. To obtain meaningful results, the membrane microstructure must be very well defined and reproducible between samples with little variation across the membrane surface. Thanks to substantial investments over the past 25 years, supported membranes have become available with a homogeneous thickness of 10–500 nm and a <1 nm local surface roughness [5,33,34,35]. The deposition of these membranes is possible due to the development of homogeneous membrane supports with a surface roughness of ~25 nm. They are made by casting a mono-sized nanoparticle dispersion on the supports, followed by thermal processing. The initial densely packed nanoparticle layer is formed by “slip-casting” in which the dispersion medium is drawn into the support by capillary action. This mechanism also ensures a very smooth membrane top surface. Thermal processing consists of a drying step followed by a high-temperature calcination step to remove solvents and additives and to form the target membrane phase. An example of membrane structure obtained as described is provided in Figure 1. In [36], we introduced and used spectroscopic ellipsometry (SE) as a non-destructive, non-contact method to determine membrane thickness and porosity. This method requires a near-optical quality structure and can only provide accurate results by measuring membrane structures as indicated.

We anticipated that by using SE, we would be able to resolve the very slow dissolution and densification of a supported mesoporous membrane material. To demonstrate the use of this method, we made membranes with presumed stable compositions and exposed them to water and dilute aqueous solutions at temperatures up to 80 °C for up to 6 weeks. We characterized these membranes using SE at intervals to determine significant changes.

In the work presented herein, we used membranes made with precursor nanoparticle dispersions for (1) TiO_2_ anatase using an alkoxide hydrolysis method; (2) cubic CeO_2_, yttria-stabilized zirconia (YSZ) and Fe_3_O_4_ (magnetite) made via sonochemical precipitation; and (3) amorphous SiO_2_, which was obtained as a commercially available dispersion. The dispersions were adjusted for deposition properties by adding polyvinyl alcohol (PVA). Subsequently, thin particle layers were formed on smooth, macro porous supports by dip coating. The eventual membrane composition was formed via drying and rapid thermal processing (RTP) with target temperatures of 600 to 700 °C. For the exposure experiments, we used mostly ultrapure water and occasionally performed a targeted adjustment of the pH by adding nitric acid (HNO_3_) or tetramethylammonium hydroxide (TMAOH).

## 2. Materials and Methods

Thin, supported inorganic membranes are generally made via the deposition of a stable precursor sol on macroporous α-Al_2_O_3_ supports, followed by thermal processing [6]. The deposition occurs by film coating and/or slip-casting (filtration driven by capillary suction). The sols are made by wet-chemical precipitation or polymerization methods in which well-dispersed particles are obtained, either immediately or by peptization [5,37,38]. A post-treatment of the sols to remove larger particles and agglomerates is generally needed. In addition, molecular or polymeric compounds are added to promote membrane formation. Thermal processing consists of drying, the removal of additives, the conversion of precursor phases into the target structure and partial sintering to form a coherent structure. During sintering, particles in a compact form necks and merge, driven by surface tension reduction. At least some neck formation (with little shrinkage) is needed to give the structure sufficient strength [39].

The overall membrane synthesis process used for the studies presented here includes five steps. (1) Macroporous α-Al_2_O_3_ supports are prepared, beginning with the colloidal casting of commercially available particles. (2) Precursor dispersions containing nanosized particles are synthesized by alkoxide hydrolysis or sonochemical precipitation methods or obtained commercially. (3) Supported membrane precursors are made by dip-coating mixtures of precursor dispersions and additives such as a binder and lubricant (PVA) on α-Al_2_O_3_ supports. (4) Most of the water and some additives are removed by drying the samples in an oven. (5) The final oxide structure is obtained with rapid thermal processing (RTP).

### 2.1. Support Synthesis

Macroporous, disk-shaped α-Al_2_O_3_ supports with optically smooth deposition surfaces were made according to a procedure described in [6]. Stable dispersions of 50 wt% α-Al_2_O_3_ powder (AKP30, Sumitomo Chemical, Tokyo, Japan) in 0.01 M aqueous HNO_3_ (Sigma Aldrich, St. Louis, MO, USA) were prepared by ultrasonification (Branson Utrasonics, Brookfield, CT, USA), followed by mesh screening (20 µm) and the removal of microbubbles using biaxial centrifugation (Thinky U.S.A., Laguna Hills, CA, USA). The supports were formed by vacuum filtration. After drying in the filtration mold for ~24 h, the consolidates (“filter cakes”) were transferred into an alumina crucible boat (Fisher Scientific, Pittsburgh, PA, USA) and sintered at 950 °C for 10 h using heating and cooling rates of 2 °C/min. The obtained α-Al_2_O_3_ disks had a diameter of 42 mm, a thickness of about 2 mm, a porosity of 35% and bulk pore size of 100 nm, as determined by mercury porosimetry (Micromeritics, Norcross, GA, USA), a surface pore size of 40 nm and a surface roughness determined using permeation porometry [40].

### 2.2. Nanoparticle Dispersion Syntheses

#### 2.2.1. Titania Dispersion Synthesis

The titania dispersion was made by starting from the hydrolysis of titanium (IV) isoproproxide. Ultrapure de-ionized (DI) water (0.056 µS/cm; Merck Millipore, Burlington, MA, USA) was combined with HNO_3_ at a 40:1 molar ratio. While the solution was stirred at 50 °C, a mixture of titanium (IV) isopropoxide and isopropanol (Thermo Scientific Chemicals, Waltham, MA, USA) was added via a syringe and syringe pump at a rate of 150 mL/h. The use of a syringe prevented the air exposure of the alkoxide. The titania dispersion, thus obtained, was centrifuged at 25,000 rpm for 3 h (Allegra 64R; Beckman Coulter, Brea, CA, USA). Immediately after centrifugation, 10 mL of the supernatant was stored for use in membrane deposition.

#### 2.2.2. Sonochemical Precipitation

Homogeneous ceria precursor dispersions were synthesized in four steps. (1) A solution of cerium ammonium nitrate ((NH_4_)_2_Ce(NO_3_)_6_ (Sigma Aldrich, St. Louis, MO, USA) was treated with intense ultrasound waves generated with an ultrasonic probe operating at 20 kHz and with a power of 20–35 W. Microbubbles formed in the tensile phase of the ultrasound and collapsed quickly in the compression phase, causing local temperatures of up to 5000 K. This resulted in the formation of isolated and insoluble nuclei at the vanishing points of the bubbles. (2) TMAOH (Sachem, Austin, TX, USA) was added to form larger particles from the nuclei by precipitation. (3) N,N-Bis(2-hydroxyethyl) glycine (Bicine, Sigma Aldrich, USA) was added to suppress any agglomeration that would adversely affect subsequent processing. Due to the nature of this process, the nanoparticles in the eventual dispersion did not agglomerate and had a narrow size distribution within 10 nm. (4) After sonication, dialysis was performed to remove excess ions in turn to avoid the precipitation of salts during later processing and to further improve dispersion stability. An aqueous solution of 1 N HNO_3_ (Sigma Aldrich, USA) with a pH of 2 was used as a dialysate.

YSZ and magnetite nanoparticle precursor dispersions were also prepared using similar sonochemical precipitation methods. For the YSZ synthesis, an aqueous solution of yttrium nitrate hexahydrate (Y(NO_3_)_3_·6H_2_O, Sigma Aldrich, USA) and zirconium oxynitrate hydrate (ZrO(NO_3_)_2_·xH_2_O, Sigma Aldrich, USA) was used as a precursor in step 1. A magnetite dispersion was made using an aqueous iron nitrate (3-hydrate) solution in step 1.

### 2.3. Dynamic Light Scattering

The particle size distributions of the synthesized nanoparticle dispersions were measured using Dynamic Laser Scattering (DLS; Malvern Panalytical, Malvern, UK). In DLS diffusion, coefficients of Brownian motion are obtained which, in turn, are used to obtain particle size distributions from the Stokes–Einstein equation [41].

### 2.4. Stability Analysis

The stability of inorganic mesoporous membranes can be studied by observing microstructural changes, changes in transport properties and by analyzing trace ions in solutions that have been in contact with the membrane material. However, as will be shown in this study, the dissolution rates of “stable” membranes can be as small as 1 nm over 8 weeks (2 × 10^−16^ m/s). At such low dissolution rates, any changes cannot be observed with normal, destructive microstructure characterization methods. In transport characterizations, the membrane must be kept in the module to obtain sufficient reproducibility. However, even clean water characterizations are dominated by residual fouling with module components, while no clear distinction can be made between thickness and pore size effects. A dissolution rate of 2 × 10^−16^ m/s for TiO_2_, with a membrane surface of 1.4 × 10^−3^ m^2^, as we use, and a water volume of 1 L would cause a Ti^4+^ concentration change of 7 × 10^−8^ mol/L. This is close to the ICP-OES detection limit of 10^−7^ mol/L and above the ICP-MS detection limit of 2 × 10^−9^ mol/L. However, contamination from the ambient and liquid, initially and over time, is likely to exceed those values by orders of magnitude [42]. In addition, the analysis of the liquid is still an indirect method.

### Spectroscopic Ellipsometry

In spectroscopic ellipsometry (SE), changes in the polarization of light reflected from a sample surface are analyzed to obtain the properties of single- and multi-layer structures, such as their thickness, refractive index and optical absorption and the anisotropy of and gradients in those properties [43]. Ellipsometry measurements can also be performed in situ to monitor membrane formation and adsorption [44,45]. The change in polarization after reflection is expressed in terms of the intensity, *r*_s_, of light, with polarization perpendicular to the incidence plane, and the intensity, *r*_p_, with polarization parallel to the incidence plane. Measured values of *r*_p_ and *r*_s_ are then used to obtain Ψ and Δ in

(1) $$\tan\left(\Psi \right)\exp\left(i\Delta \right)=r_{p}/r_{s}$$

where tan(Ψ) is the amplitude ratio between reflected light with p- and s-polarizations and Δ is the phase difference between reflected light with p- and s-polarizations [46]. SE measurements were performed for every sample before and after each stability test using a VASE ellipsometer (J.A. Woollam, Lincoln, NE, USA), with which Ψ and Δ were obtained for three incident angles, 65, 70 and 75°, in a wavelength range of 300 to 1500 nm. Prior to the experiment, the samples were marked such that the data were always obtained for the same location.

The SE data were analyzed using J.A. Woollam’s WVASE v3.934 software. Since the membranes were mostly transparent and colorless at the wavelengths of observation, the Cauchy model was used for the wavelength dependence of the refractive index.

(2) $$n\left(\lambda \right)=A+\frac{B}{\lambda^{2}}+\frac{C}{\lambda^{4}}$$

where *n* is the refractive index, λ is the wavelength and *A*, *B* and *C* adjustable parameters. Best fits were obtained by the minimization of the non-linear Mean Square Error [46]. Precisions estimated were obtained assuming a normal distribution with a 95% confidence interval.

The following effective characteristics were obtained, moving from the membrane surface into the support:

- The roughness of the exposed membrane surface was typically within 1 nm.
- A membrane thickness in the range of 10 nm and 1 μm, with precision and variation of the membrane surface of ±1 nm.
- An effective thickness of intermixing between the membrane and the support of the order of 50 nm for supports with a surface pore size of ~40 nm and a short-range surface roughness of ~25 nm [6,40].
- A refractive index of 1.5 to 2 ± 0.05 that can be used for an accurate estimate of porosity through the Bruggeman method [36].

## 3. Results and Discussion

All nanoparticle dispersions appeared visually transparent. The titania precursor dispersion made by alkoxide hydrolysis and centrifugation had a particle size distribution of 1 to 4 nm with an average of 2.3 nm. The sonochemical precipitation syntheses resulted in precursor dispersions with a size distribution of 2 to 3.5 nm and an average size of 2.8 nm for YSZ and a size distribution of 4 to 8 nm with an average of 4.2 nm for ceria. The as-received silica dispersion (Ludox AS, Sigma-Aldrich) had a particle size distribution of 4 to 6.4 nm with an average of 5.2 nm. The membranes made with these dispersions had thicknesses of 100 to 300 nm and showed opalescent effects that are characteristic of thin, homogeneous, transparent membranes. The titania, zirconia, ceria, magnetite and silica membranes appeared light blue, light yellow, orange, dark brown and colorless, respectively. No delamination from the supports or micron-scale roughness was observed.

**Figure 2:**
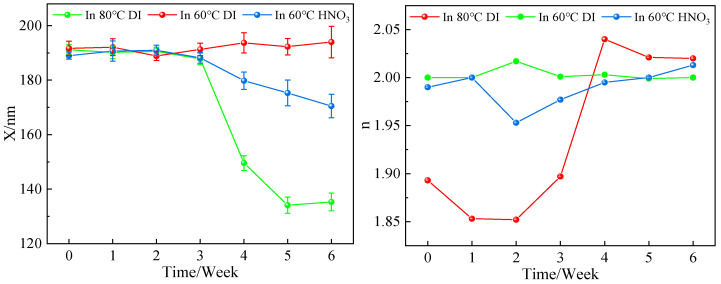
Results of stability measurements for ceria membranes.

**Figure 3:**
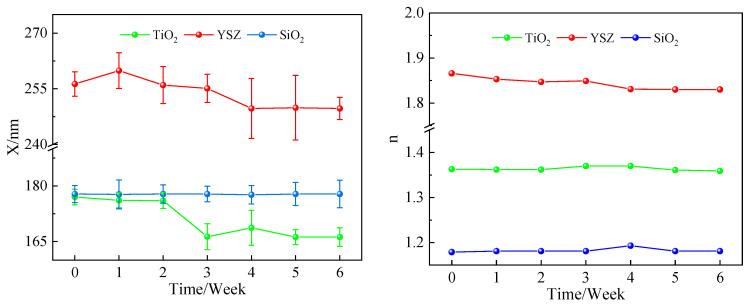
Results of stability measurements for titania, YSZ and amorphous silica membranes in 80 °C DI.

The results of the stability characterizations are presented in Table 1, Table 2, Table 3 and Table 4. Graphical representations of the most important results are provided in Figure 2 and Figure 3.

It was found that the variation in the thickness of a membrane across the membrane’s surface within a radius of 1.5 mm was typically 1 nm. The difference in membrane thickness between samples made in similar ways was within 10 nm. An analysis of the stability data indicated that the dissolution rate could be resolved within 0.1 nm/week and the densification rate within 0.1%/week.

Except for amorphous silica and YSZ, none of the other oxides appeared to be fully water stable at 80 °C within the limitations of observation. Below is a summary of the findings thus far, including results from the literature for γ-alumina.

- Mesoporous γ-alumina made through the peptization of hydrolyzed aluminum-tri-sec-butoxide (ATSB) typically dissolves in aqueous solutions of any pH within 24 h [30,31,32]. One way to suppress this is to add a substantial number of Al^3+^ ions to the solution [31], but this approach cannot be used for most applications.
- Mesoporous amorphous SiO_2_ made from Ludox AS dispersions appeared to be fully stable, within the limits of observation, in ultrapure water at a temperature of 80 °C.
- Crystalline CeO_2_ membranes made through sonochemical precipitation were stable in ultrapure water at 60 °C, but at 80 °C, their thickness decreased at a rate of 11.5 nm/week while they densified at a rate of 4.6%/week. The membranes dissolved at a rate of 3.4 nm/week and densified at a rate of 1.1%/week in 0.01M aqueous HNO_3_ at 60 °C.
- Cubic zirconia (YSZ) made through sonochemical precipitation was quite stable, with a minor dissolution rate of 1.6 nm/week in ultrapure water at 80 °C. Meanwhile, its porosity increased at a rate of 0.4%/week during the test period, indicating that a slight dissolution at 80 °C results in an increased membrane porosity.
- Magnetite Fe_3_O_4_ was stable in an aqueous solution of TMAOH with a pH of >11. It quickly dissolved in aqueous solutions of nitric acid with pH values of <4. This is expected since iron oxides are known to dissolve quickly at low pH values.
- Anatase TiO_2_ made via alkoxide hydrolysis was stable in ultrapure water at 80 °C for 2 weeks. Then, the thickness decreased by 10 nm during week 3 but did not change from week 4 to week 6. The refractive index hardly changed (±0.01) over 6 weeks. The thickness decrease is significant and is tentatively ascribed to a transition to a more stable TiO_2_ phase.

During the experiments, the ultrapure water likely became CO_2_-buffered due to the dissolution of CO_2_ from ambient air. Such buffering leads to pH = 6.8 at room temperature, 6.5 at 60 °C and 6.2 at 80 °C. In addition, some Na^+^ and borate ions may have been released from the glass container. We believe that neither effect is of any significance for the water stability experiments conducted.

The most stable oxides appear to be those with (1) cations that exist solely in the 4^+^ form and (2) that are in their most stable phase. Si ions occur only in the 4^+^ state and form strong covalent Si-O bonds in a tetrahedral coordination. CeO_2_ and TiO_2_ can be slightly reduced with the formation of minor concentrations of Ce^3+^ and Ti^3+^ with charge compensation by oxygen vacancies. Ce^3+^ and Ti^3+^ are more accurately described in terms of free electrons in the conduction band. Both vacancies and electrons make the oxides more accessible for reaction with H_3_O^+^ and OH^−^. The Y^3+^ in YSZ can segregate and leach out. However this phenomenon is known to occur only at larger particle sizes of >400 nm [47], while the grain size in our membranes was ~5 nm [48]. The Y^3+^ is added to obtain the cubic zirconia structure, but the cubic symmetry is also stabile at very small grain sizes. Even if the Y^3+^ leaches out, the zirconia scaffold may remain intact. The consideration of an exclusive 4+ state and the formation of strong bonds with oxygen makes it unlikely that oxides of other elements will ever be sufficiently stable. PrO_2_ would be similar to CeO_2_ but it tends to stabilize into Pr_6_O_11_ with substantial Pr^3+^ which, in turn, results in too much solubility, as we confirmed in a quick test. Except for γ-alumina and silica, the oxides studied form no anions at pH > 7. Silica only forms silicates in solutions at very high pH values and with prolonged treatment. Since Fe_3_O_4_ appeared very unstable at low pH values and contains 2+ and 3+ ions, we conducted a test at pH = 11.

In the solid state, γ-alumina is not a stable against the formation of α-Al_2_O_3_ at high temperatures. Amorphous silica is normally metastable up to 1400 °C; slow formation of the stable cristobalite phase occurs at temperatures >1000 °C but must promoted by Na^+^ ions. CeO_2_, YSZ and Fe_3_O_4_ form a stable cubic phase from the precursor via calcination at moderate temperatures. Anatase TiO_2_ is not stable with respect to the rutile phase, which forms around 600 °C.

One truly remarkable result is the combination of the complete stability of amorphous silica in water up to 80 °C and the factual complete instability of γ-alumina. Microporous amorphous silica membranes are well known for their high-flux, highly selectivity separation of light molecules [49]. However these membranes are reported to be unstable in humid air of ~20% relative humidity at 100 °C [50]. On the other hand, the microporous amorphous silica membranes reported thus far are mostly made via the modification of supported γ-alumina. Consequently, we speculate that the instability may have been caused by the degradation of the γ-alumina membrane rather than the silica modification. To our knowledge, the only other type of mesoporous scaffold is mesoporous silica made by surfactant-assisted self-organization, but no stability results have been reported for those membranes [51,52]. Hence, we recommend investigation of the performance of microporous amorphous silica membranes with scaffolds made of amorphous silica, ceria, zirconia, titania, magnetite or other water-stable oxides.

## 4. Conclusions

It was found that anatase titania commonly used and proposed and γ-alumina membranes are not completely stable in water at elevated temperatures. This raises concerns about their actual long-term water stability in any conditions. Amorphous mesoporous silica is not widely explored for water purification but appears to be much more stable. Since the intended use of mesoporous inorganic membranes is in large-scale water treatments, the results and methods provided in this work are deemed essential to make better choices for membrane compositions that are developed. The fact that SE can be conducted in situ, under actual process conditions, can be beneficial for obtaining the most relevant stability information within a limited time frame.

Homogeneous, thin, mesoporous amorphous silica, ceria, titania, YSZ and magnetite membranes can be synthesized for demonstrating the use of spectroscopic ellipsometry in water stability studies. It was possible for all these membranes to obtain thickness and refractive index values with precision of 1 nm and 0.001 and rates of change as small as 0.1 nm/week and 0.001/week, respectively. However, the results may not be fully representative of any mesoporous oxide membrane with a similar composition. The total duration of the work presented was about a year. A systematic investigation of the stability of just one oxide with the membrane made using one particular route in a range of practical solution compositions may take much more time and effort, yet we believe that the trends in stability shown herein are significant.

The water-stable oxides identified in this work all have 4+ charges. Hence, other interesting systems to investigate include GeO_2_, SnO_2_ and PbO_2_, but these are less likely to be stable because of lower bond strengths. The observed densification behavior of ceria membranes and increases in porosity in YSZ membranes may be utilized to achieve membranes structures that cannot be otherwise obtained.

## Figures and Tables

**Table 1:** Results of stability measurements for ceria membranes.

	In 80 °C DI		In 60 °C DI		In 60 °C HNO_3_	
Week	*X* (nm)	*n*	*X* (nm)	*n*	*X* (nm)	*n*
0	191.2 ± 1.8	1.893 ± 0.001	191.7 ± 2.6	2.000 ± 0.001	189.0 ± 1.3	1.990 ± 0.001
1	190.2 ± 2.3	1.853 ± 0.001	192.1 ± 3.1	2.000 ± 0.001	190.7 ± 3.7	2.000 ± 0.001
2	190.6 ± 1.5	1.852 ± 0.001	188.9 ± 1.7	2.017 ± 0.001	191.0 ± 1.8	1.953 ± 0.001
3	187.8 ± 2.1	1.897 ± 0.001	191.3 ± 2.3	2.001 ± 0.001	188.2 ± 2.0	1.977 ± 0.001
4	149.6 ± 2.7	2.040 ± 0.002	193.7 ± 3.7	2.003 ± 0.001	179.8 ± 3.2	1.995 ± 0.001
5	134.1 ± 3.0	2.021 ± 0.001	192.3 ± 3.0	1.999 ± 0.001	175.3 ± 4.7	2.000 ± 0.001
6	135.3 ± 3.3	2.020 ± 0.001	194.0 ± 5.8	2.000 ± 0.001	170.5 ± 4.3	2.013 ± 0.002

**Table 2:** Results of stability measurements for titania, YSZ and amorphous silica membranes in 80 °C DI.

	TiO_2_		YSZ		SiO_2_	
Week	*X* (nm)	*n*	*X* (nm)	*n*	*X* (nm)	*n*
0	177.0 ± 2.1	1.363 ± 0.001	256.3 ± 3.3	1.866 ± 0.001	177.8 ± 2.3	1.179 ± 0.001
1	176.1 ± 2.0	1.362 ± 0.001	259.9 ± 4.8	1.853 ± 0.001	177.7 ± 3.9	1.181 ± 0.001
2	176.0 ± 2.1	1.362 ± 0.001	256.0 ± 5.0	1.847 ± 0.001	177.8 ± 2.5	1.181 ± 0.001
3	166.3 ± 3.5	1.370 ± 0.001	255.1 ± 3.8	1.849 ± 0.001	177.8 ± 2.1	1.181 ± 0.001
4	168.7 ± 4.7	1.370 ± 0.002	249.7 ± 8.1	1.831 ± 0.001	177.6 ± 2.5	1.193 ± 0.001
5	166.2 ± 2.0	1.361 ± 0.001	249.9 ± 8.7	1.830 ± 0.002	177.8 ± 3.1	1.181 ± 0.001
6	166.2 ± 2.5	1.359 ± 0.001	249.7 ± 3.0	1.830 ± 0.001	177.8 ± 3.7	1.181 ± 0.001

**Table 3:** Results of 24 h stability measurements for magnetite membranes.

Solution and pH	Before *X* (nm)	After *X* (nm)	Before *n*	After *n*
TMAOH @ 11	258.3 ± 3.9	253.7 ± 5.3	4.623 ± 0.001	5.147 ± 0.001
HNO3 @ 5	253.7 ± 5.3	265.1 ± 4.6	5.147 ± 0.001	3.785 ± 0.001
HNO3 @ 4	265.1 ± 4.6	216.6 ± 7.5	3.785 ± 0.001	6.834 ± 0.002
HNO3 @ 3	216.6 ± 7.5	197.3 ± 8.3	6.834 ± 0.002	6.346 ± 0.002

**Table 4:** Membrane dissolution and densification rates calculated from measurements.

Membrane	Condition	Dissolution Rate (nm/Week)	Densification Rate (%/Week)
CeO_2_	In 80 °C DI	11.5 ± 1.3	4.6 ± 0.05
	In 60 °C DI	0.0 ± 0.4	0.0 ± 0.02
	In 60 °C at pH = 2	3.4 ± 0.7	1.1 ± 0.03
TiO_2_	In 80 °C DI	2.1 ± 0.5	0.0 ± 0.02
YSZ	In 80 °C DI	1.6 ± 0.4	−0.4 ± 0.02
SiO_2_	In 80 °C DI	0.0 ± 0.0	0.1 ± 0.00

## Data Availability

Data contained within the article.

## Abbreviations

*f*_*ℓ*_	Mechanical permeance: *f*_*ℓ*_ = *j_V_* *× η_ℓ_*/Δ*p*
*f_V_*	Volumetric permeance: *f_V_* = *j_V_*/Δ*p*
*j_V_*	Volumetric flux
*η_ℓ_*	Liquid viscosity
Δp	Mechanical pressure difference
CNT	Carbon nanotube
PZT	Lead zirconate titanate (PbZr_0.52_Ti_0.48_O_3_)
AKP30	Sumitomo Chemical AKP30 α-Al_2_O_3_ powder
GO	Graphene oxide
DI	De-ionized
YSZ	Yttria-stabilized zirconia
DLS	Dynamic Laser Scattering
TMAOH	Tetramethylammonium hydroxide
PVA	Polyvinylalcohol
RTP	Rapid Thermal Processing
SE	Spectroscopic Ellipsometry
*r_s_*	Intensity of light with polarization perpendicular to the incidence plane
*r_p_*	Intensity of light with polarization parallel to the incidence plane
tan(Ψ)	Amplitude ratio between reflected light with p- and s-polarizations
Δ	Phase difference between reflected light with p- and s-polarizations
*n*	Refractive index
λ	Wavelength
*X*	Membrane (layer) thickness
